# The effects of dialysate acids and divalent ions on calcification and cardiovascular outcomes: old wisdom and new developments

**DOI:** 10.1093/ckj/sfag313

**Published:** 2026-09-15

**Authors:** Jeroen P Kooman, Karlien J ter Meulen, Peter Kotanko, Bernard Canaud

**Affiliations:** Division of Nephrology, Department of Internal Medicine, Maastricht University Medical Center, Maastricht, the Netherlands; NUTRIM Research Institute, Maastricht University, Maastricht, Netherlands; Division of Nephrology, Department of Internal Medicine, Maastricht University Medical Center, Maastricht, the Netherlands; NUTRIM Research Institute, Maastricht University, Maastricht, Netherlands; Renal Research Institute, New York, USA; Icahn School of Medicine at Mount Sinai, New York, USA; School of Medicine, Montpellier University, Montpellier, France

**Keywords:** calcification, citrate, citric acid, dialysate calcium, dialysate magnesium

## Abstract

Dialysate calcium (DCa) and magnesium (DMg) and the organic acid used in the acid concentrate have a profound combined influence on intradialytic mineral balance and may influence vascular calcification progression and treatment-related symptoms. Whereas Kidney Disease: Improving Global Outcomes recommends a DCa between 1.25 (DCa 1.25) and 1.50 mmol/l (DCa 1.50), the optimal DCa for an individual patient remains uncertain.

No consistent mortality difference between DCa 1.50 and 1.25 has been observed. However, DCa 1.50 may induce a positive calcium balance, raising concerns about vascular calcification progression. Conversely, DCa below 1.25 mmol/l has been associated with a higher risk of sudden cardiac death. Compared with acetic acid (acetate dialysate, Acet-D), citric acid (citrate dialysate, Cit-D) reduces calcification propensity of serum, although the effect on vascular calcification progression is inconclusive.

Whenever feasible, DCa should be individualized, ideally using estimates of calcium mass balance (CaMB). Because Cit-D generally results in a more negative CaMB than Acet-D solutions at the same nominal DCa, this difference should be considered when prescribing DCa. Increasing DMg from 0.50 to 0.75 mmol/l may improve surrogate cardiovascular outcomes although its effect on clinical outcomes remains to be defined.

Overall, Acet-D with DCa 1.25 is a reasonable standard especially when using calcium-containing phosphate binders and in patients at risk for adynamic bone disease, whereas in patients with a high risk of sudden cardiac death, frequent intradialytic hypotension or with severe secondary hyperparathyroidism, DCa 1.50 may be a reasonable alternative. Regarding the choice of acid, the use of Cit-D represents a promising alternative pending further clinical evidence.

## INTRODUCTION

Chronic kidney disease-mineral and bone disorder (CKD-MBD) is highly prevalent in patients undergoing hemodialysis (HD). Vascular and valvular calcifications, largely driven by CKD-MBD, are the most prominent phenotypic manifestations of advanced kidney failure and are strongly associated with mortality. Rather than representing passive hydroxyapatite deposition, vascular calcification is now recognized as an active and tightly regulated biological process, resulting from an imbalance between pro-calcifying factors and a downregulation in endogenous calcification inhibitors. The impact of extracorporeal kidney replacement therapy on vascular calcification is more complex. On the one hand, HD and hemodiafiltration (HDF) may reduce calcification propensity through phosphate removal [1]. On the other hand, the composition of the dialysate, in the acid (A) concentrate, particularly its calcium and magnesium concentrations as well as the choice of the organic acid, may exert important effects. Excessive calcium loading may promote vascular calcification, whereas magnesium depletion may impair the endogenous defense against calcification. The intradialytic balance of ionized calcium and magnesium is influenced by dialysate concentrations but also by several patient-related factors, including total plasma protein bindings (albumin), acid–base status, parathyroid hormone (PTH), concomitant medications, and state of bone turnover. The choice of acidifying agent, most commonly acetate or citrate, also modulates calcium and magnesium kinetics, calcium mass balance (CaMB), calcification propensity, and potentially cardiovascular outcomes. The combined effects on dialysate calcium, magnesium and acidifying agents on vascular calcification and cardiovascular risk have not been comprehensively reviewed in recent years.

The aim of this narrative review is to examine the role of divalent ions and acidifying agents in the dialysate A concentrate and to discuss their effects on calcium and magnesium homeostasis, vascular calcification, calcification propensity, and cardiovascular outcomes in patients receiving HD and HDF.

## PRODUCTION OF DIALYSATE AND COMPOSITION OF THE ACID CONCENTRATE

Dialysate is prepared from three components: an A concentrate containing electrolytes, glucose, and an acidifying agent; a bicarbonate (B) concentrate containing sodium bicarbonate [2]; and purified water. This production process is commonly referred to as a three-stream, dual concentrate system [3] (Fig. 1). Prior to mixing with water, the A and B concentrates must remain physically separated because bicarbonate reacts with calcium and magnesium under alkaline conditions, resulting in precipitation of calcium and magnesium carbonates [4].

**Figure 1:**
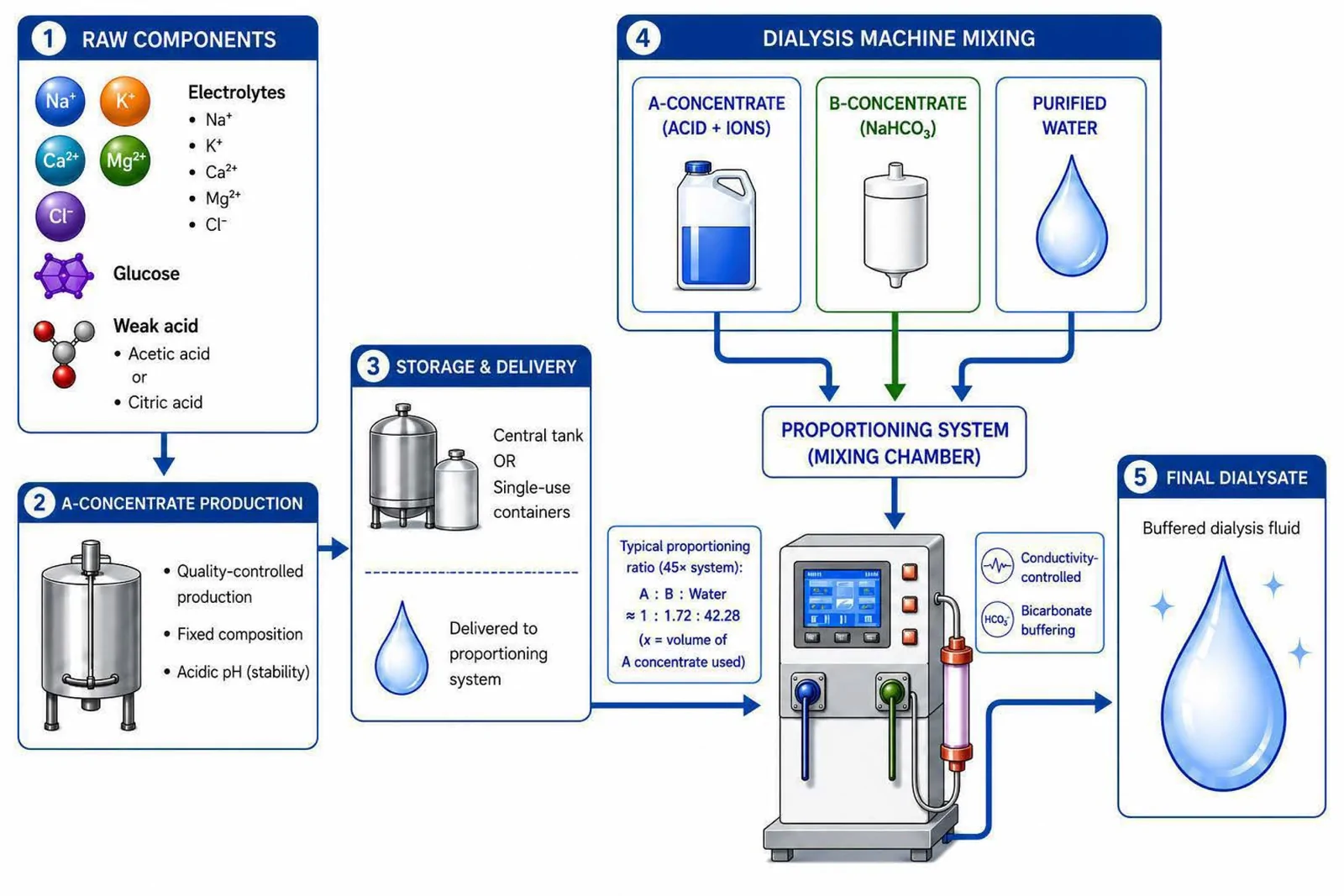
Production of bicarbonate-based dialysis fluid and composition of the A concentrate.

Within the dialysis proportioning module, the three components are mixed in a fixed volumetric ratio, e.g. 1 part A concentrate, 1.72 parts of B concentrate, and 42.28 parts of purified water in the conventional 45x system, where “45x” denotes the dilution factor of the A concentrate. The A concentrate is generally supplied as a liquid form, although dry formulations are also available. In contrast, the B concentrate is typically supplied as a dry powder cartridge. The A concentrate may be supplied either through a centralized distribution system or as a single use disposable container. Sodium concentration can be individualized by the dialysis machine through servo-controlled adjustments of the proportioning ratio based on online conductivity measurements, and a similar approach is used for bicarbonate prescription. In contrast, the concentrations of calcium, magnesium, potassium, and the acidifying agent are fixed by the composition of the A concentrate itself and can only be modified by selecting a different concentrate formulation. The acidifying agent maintains the concentrate at an acidic pH, preventing precipitation of calcium and magnesium salts during storage and mixing [4]. The A concentrate may exert important physiological effects by influencing calcium and magnesium kinetic, CaMB, and potentially cardiovascular outcomes. For these reasons, the A concentrate should not be regarded simply as a technical component of dialysis fluid preparation but as an integral part of the dialysis prescription (Fig. 1).

Acetic acid is the most used acidifying agent. During dialysate preparation, acetic acid is buffered by the bicarbonate (B) according to the reaction:


\begin{eqnarray*}
{\mathrm{NaHC}}{{\mathrm{O}}_3} + {\mathrm{C}}{{\mathrm{H}}_3}{\mathrm{COOH}}\rightarrow{{\mathrm{H}}_2}{\mathrm{C}}{{\mathrm{O}}_3} + {\mathrm{C}}{{\mathrm{H}}_3}{\mathrm{COONa}}\rightarrow \\{\mathrm{C}}{{\mathrm{O}}_2} + {{\mathrm{H}}_2}{\mathrm{O}} + {\mathrm{C}}{{\mathrm{H}}_3}{\mathrm{COONa}}
\end{eqnarray*}

The generated carbon dioxide is eliminated through respiration, while acetate is subsequently metabolized to bicarbonate, primarily in the liver and skeletal muscle [5]. Because bicarbonate consumed during neutralization is regenerated through acetate metabolism, the addition of the small amount of acetic acid (typically ≈3.0 mmol/l) to the A concentrate does not result in a net gain or loss of buffer. In some formulations, acetate is supplied as sodium diacetate, a compound consisting of equimolar acetic acid and sodium acetate.

Importantly, this situation must not be confused with the historical “acetate dialysis” used before the widespread adoption of “bicarbonate dialysis” in the 1980s. In “acetate dialysis,” acetate served as the principal buffer and was administered at much higher concentrations, relying on systemic metabolism through acetyl coenzyme A to generate bicarbonate. The low acetate concentrations currently used in bicarbonate dialysis serve primarily as a pH stabilizer rather than as the main buffering agent.

A similar buffering reaction occurs when citric acid is used instead of acetic acid as pH stabilizer, resulting in citrate formation as the organic anion:


\begin{eqnarray*}
{{\mathrm{C}}_6}{{\mathrm{H}}_8}{{\mathrm{O}}_7} + 3{\mathrm{NaHC}}{{\mathrm{O}}_3}\rightarrow{\mathrm{N}}{{\mathrm{a}}_3}{{\mathrm{C}}_6}{{\mathrm{H}}_5}{{\mathrm{O}}_7} + 3{\mathrm{C}}{{\mathrm{O}}_2} + 3{{\mathrm{H}}_2}{\mathrm{O}}
\end{eqnarray*}

Citric acid is added to the A concentrate in an amount yielding ∼0.8–1.0 mmol/l citrate in the final dialysate (corresponding to 2.4–3.0 mEq/l as citrate is a trivalent ion). Besides maintaining solution acidity, citrate partially chelates ionized calcium and magnesium, thereby modifying their intradialytic kinetics and CaMB. Citrate-containing bicarbonate dialysis must not be confused with regional citrate anticoagulation used during continuous renal replacement therapies or regional anticoagulation in intermittent HD [6]. In regional citrate anticoagulation, citrate is infused at substantially higher concentrations to chelated calcium within the extracorporeal circuit, providing anticoagulation while also serving as a bicarbonate precursor through cellular metabolism. Throughout this review, bicarbonate dialysis using an acetate-containing A concentrate will be referred to as acetate dialysate (Acet-D), whereas bicarbonate dialysis using a citrate-containing A concentrate will be referred to as citrate dialysate (Cit-D).

Despite considerable interindividual variability in mineral metabolism among patients receiving HD, many HD facilities continue to prescribe a standardized A concentrate for most patients. Although centralized delivery system simplifies logistics and routine clinical operations, individualized A concentrates remain operationally more demanding. Nevertheless, the lack of individualization may have important physiological consequences, as the A concentrate should be considered as a potent therapeutic component of the dialysis prescription rather than merely a medical device solution. The following sections review the clinical implications and pathophysiological basis of the A concentrate, with particular emphasis on its calcium, magnesium, and acidifying agent composition.

## CALCIUM

Dialysate calcium concentration (DCa) is a key determinant of calcium homeostasis during HD and remains one of the most debated components of the dialysis prescription. The two concentrations (DCa) most used in clinical practice are 1.25 and 1.50 mmol/l (equivalent to 2.5 and 3.0 mEq/l). The current Kidney Disease: Improving Global Outcomes (KDIGO) guidelines recommend either concentration, allowing flexibility for individualized clinical decision-making [7]. The optimal prescription should be interpreted in relation to the baseline predialysis ionized calcium concentration, which largely determines the transmembrane calcium gradient and consequently intradialytic calcium flux and overall CaMB.

### Clinical outcomes

The clinical consequences of different DCa prescriptions remain controversial. DCa below 1.25 mmol/l have been associated with an increased risk of sudden cardiac death (SCD) [8], whereas a DCa of 1.75 mmol/l (DCa 1.75) has been linked to accelerated vascular calcifications and increased mortality [9].

Evidence comparing the two concentrations currently recommended by KDIGO (1.25 versus 1.50 mmol/l) is considerably less convincing. A recent systematic review and meta-analysis including 19 randomized controlled trials comparing lower DCa (1.25 or 1.125 mmol/l) with higher DCa (1.50 or 1.75) found no significant differences in either all-cause or cardiovascular mortality. However, most studies were small and not powered for mortality endpoints, limiting the strength of their conclusions [10]. Similarly, the REIN registry reported no mortality difference between patients treated with DCa ≤1.50 and >1.50 [11].

Large observational studies have yielded inconsistent findings. In a recent analysis including 39 886 patients followed for 2 years, DCa 1.50 was associated with higher all-cause and cardiovascular mortality in unadjusted analyses; however, these associations disappeared after adjustments. In contrast, the adjusted incidence of SCD was higher with DCa 1.25 [12]. An observational study in 1182 incident HD patients followed for a median of 16 months reported increased mortality with DCa >1.50, but no difference between DCa 1.25 and 1.50 [13]. Data from the DOPPS cohort also suggested an increased mortality risk with higher DCa levels, although a direct comparison between DCa 1.25 and 1.50 was not specifically performed [14] Conversely, the Japanese DOPPS including 9201 patients followed for ∼2 years, found no significant differences in outcomes among DCa 1.25, 1.375, and 1.50, neither before nor after adjustment [15].

A subanalysis of the EVOLVE trial including 3883 patients, showed no significant differences in (cardiovascular) mortality or major cardiovascular events across DCa categories ranging from <1.25 to >1.50 mmol/l [16] although cinacalcet was associated with a lower risk of the primary endpoint in patients treated with DCa >1.25. Cinacalcet may modify intradialytic calcium kinetics indirectly by lowering ionized calcium and bone turnover, thereby altering the serum-to-dialysate calcium gradient and, theoretically, the intradialytic exchange between plasma and rapidly exchangeable skeletal calcium pool. Although hypothesis generating, these observations further support considering DCa in the context of the overall CKD-MBD treatment strategy rather than as an isolated prescription variable.

### Vascular calcification and hemodynamic effects

Studies examining the impact of DCa on vascular pathology have produced conflicting results. In theory, lower DCa can reduce vascular calcification progression by reducing the calcium load but can also aggravate secondary hyperparathyroidism [17]. Elevated levels of PTH have been associated with an increased risk of vascular calcification, although this relation is likely mediated by phosphate burden and bone turnover [18]. A higher DCa can increase calcium loading, whereas the extent to which this translates into vascular calcification likely depends on several modifying factors such as bone turnover and endogenous calcification inhibitors. As for the clinical evidence, in a randomized study of 128 HD patients, He *et al*. reported lower carotid intima-media thickness, reduced pulse wave velocity, and improved survival with DCa 1.25 compared with DCa 1.5 mmol/l. Most patients in that study were receiving calcium-based phosphate binders [19]. In contract, Kim *et al*., observed in a randomized study of 76 HD patients, progression of coronary artery calcification score with DCa 1.25 but not with DCa 1.50. Also in this study, most patients were receiving calcium-based phosphate binders. The authors hypothesized that secondary hyperparathyroidism and higher vitamin D doses in the low DCa group might partly explain this paradoxical finding [20].

Regarding intradialytic hemodynamic tolerance, small studies showed marked differences between DCa 1.25 and 1.75, mainly attributable to the positive inotropic effect of higher calcium concentrations [21]. Studies directly comparing DCa 1.25 and 1.50 have yielded mixed results. Gabutti *et al*., in two randomized crossover studies, reported higher nadir systolic blood pressure in patients with DCa 1.50 and reduced intradialytic hypotension in one trial [22, 23]. In contrast, Basile *et al*. found no significant hemodynamic differences between DCa 1.25 and 1.50 in stable patients [24]. Overall, due to absence of solid results from large adequately powered trials, one cannot ascertain that the prescription of a DCa 1.25 versus 1.50 has a major impact on hemodynamic response in stable patients. Nevertheless, DCa 1.50 may confer a modest benefit in selected patients prone to intradialytic hypotension [25].

### Calcium mass balance

Ideally, prescription of DCa should be guided by an estimation of total CaMB, taking into account not only intradialytic calcium transfer but also oral calcium intake (∼400–900 mg/day), reduced intestinal absorption in HD patients, vitamin D therapy, and fecal calcium losses (∼100–200 mg/day) [26, 27].

Intradialytic CaMB is highly complex. Ca circulates in blood in three forms: plasma ionized calcium (PiCa)), complexed Ca (e.g. bound to citrate or carbonate), and albumin-bound Ca [28, 29]. The diffusive component is primarily determined by the gradient between DCa and PiCa, as well as by complexed Ca species [30]. PiCa is itself influenced by dialysate bicarbonate concentrations, since alkalosis promotes binding of calcium to proteins and reduces PiCa. In addition, the Gibbs–Donnan effect, which favors retention of positively charged ions within the intravascular compartment because of negatively charged plasma proteins, must also be considered. The convective component of CaMB is mainly related to ultrafiltration volume and PiCa [31, 32]. Also in dialysate, not all calcium is present in ionized form, likely due to complexation with bicarbonate and acetate. Bech *et al*. found that Ca^2+^ in dialysate samples collected from a port just before the dialysis filter, e.g. after mixing with the B compartment, was substantially lower than the prescribed amount, i.e. 1.05 mmol/l with DCa 1.25 and 1.20 mmol/l with DCa 1.50 [33].

In the review by Basile, CaMB was consistently positive with DCa 1.75, whereas reported values with DCa 1.50 ranged between −204 ± 124 to +298 ± 132 mg, and from −578 ± 389 to −6 ± 36 mg with DCa 1.25 [34]. These discrepancies likely reflect differences in calculation methods (blood- vs dialysate-based measurements), dialysis prescriptions, and patient characteristics. In general, intradialytic CaMB is negative with DCa 1.25 [33], although some patients may still experience a positive balance at this concentration [35]. In patients treated with online HDF and DCa 1.25, mean CaMB was −309 mg with a dialysate bicarbonate prescription of 26 mmol/l and −108 mg with a bicarbonate at 32 mmol/l [36].

Given the strong dependence of CaMB on the DCa to PiCa gradient, this difference was attributed to the reduction in PiCa induced by higher dialysate bicarbonate concentrations. Overall, no major differences in CaMB appear to exist between conventional HD and online HDF [29].

PTH status can be strongly influenced by the choice of DCa [33] but can also have a substantial influence on CaMB, possibly through rapid mobilization of calcium from the bone surface through osteocalcin. In a study stratified according to PTH levels, patients with PTH >300 pg/ml, treated with DCa 1.25, exhibited a more strongly negative CaMb than patients with PTH <300 pg/ml [30]. However, another study did not observe an effect of PTH on CaMB [37].

### Estimation of calcium mass balance

A rough estimate of CaMB can be obtained by multiplying the intradialytic change in PiCa by the estimated extracellular volume (ECV) (±20% of body weight) corrected for ultrafiltration volume *[CaMB=(ΔPiCa × ECV)−(UF × *average *PiCa)]* [38]. However, the accuracy of this simplified approach is limited by several factors, including calcium complexation in both plasma and dialysate.

More sophisticated approaches, such as the single pool variable-volume kinetic model proposed by de Filippo *et al*. may provide greater precision [39]. However, the complexity of these calculations, including differential equations, limits their routine clinical applicability. Importantly, current models generally fail to account for the rapidly exchangeable calcium pool, likely located at the bone surface, which may lead to underestimation of calcium loading during dialysis. Likewise, inadequate consideration of complexed calcium removal may underestimate calcium losses. [29, 32, 39]. Timed dialysate collections remain the reference method for CaMB assessment, although they are technically challenging because even small inaccuracies may substantially affect calculations given the very large dialysis fluid volumes involved [39, 40]. Future developments, including semicontinuous calcium monitoring in fresh and spent dialysate using optical techniques, may eventually allow real-time online assessment of CaMB [41].

### Practical implications

Clinical studies commonly report predialysis PiCa levels ∼1.15 mmol/l, albeit with a fairly broad interpatient range [33, 42]. From a practical clinical perspective and given the limited commercial availability of DCa 1.375, it appears reasonable to prescribe DCa 1.25 as a standard concentration, particularly in units where calcium-containing phosphate binders are preferentially used. DCa 1.25 appears particularly preferable in patients with suspected adynamic bone disease or low PTH levels (Table 1). Some authors have advocated a DCa of 1.00 mmol/l in patients with severe vascular calcification [43], although this strategy must be balanced against the increased risk of SCD with low DCa [8]. Conversely, DCa 1.50 may be preferable in patients at high-risk of arrhythmia or SCD, those with prolonged corrected QT (QTc) intervals, in patients with severe hemodynamic instability, or in patients with severe secondary hyperparathyroidism, particularly when PiCa levels are relatively low [38, 44].

**Table 1:** Clinical implications of prescribed DCa.

DCa	Potential advantages	Potential concerns	Potential clinical indications
**<1.25 mmol/l** *Very low (e.g. 1.0 mmol/l) DCa*	Maximizes negative calcium balance May reduce vascular calcification progression	Highest risk of QT prolongation, arrhythmias, SCD, and hypotension Worsening hypocalcemia Very limited evidence; short-term use only	Cases of severe calcium overload (e.g. calciphylaxis)
**1.25 mmol/l** *Low-normal DCa*	Neutral/negative calcium balance limits calcium loading	Risk of QT prolongation, arrhythmias, and intradialytic hypotension Negative calcium balance, especially with citric acid dialysis	Appropriate when minimizing calcium exposure is desired Suspected adynamic bone disease or low PTH levels Combination with calcium-containing phosphate binders
**1.375 mmol/l** *Intermediate DCa*	Calcium balance closer to neutral Possible compromise between cardiovascular stability and calcium loading	Limited availability and limited clinical outcome data	Potential compromise between calcium loading and cardiovascular stability
**1.50 mmol/l** *High-normal DCa*	Potentially lower risk of hypocalcemia, QT prolongation, and intradialytic hemodynamic instability	Neutral to positive calcium balance May promote vascular calcification and low-turnover bone disease Increased vascular stiffness	Prolonged QTc interval Secondary hyperparathyroidism or low serum calcium Prone to intradialytic hypotension High risk or arrythmia or SCD
**1.75 mmol/l** *High DCa (restricted use)*	Maximizes calcium transfer and PTH suppression Hemodynamic stability due to inotropic effect	Markedly positive calcium balance with increased vascular calcification risk Higher mortality rate in observational studies	Close monitoring and limited time for selected patients Short term for hungry bone syndrome, symptomatic hypocalcemia, or hemodynamic instability

## THE ACIDIFYING COMPONENT

As discussed previously, it is important to distinguish acetic acid and citric acid, which are added to the dialysate A concentrate, from their respective metabolites, acetate and citrate. Before 1980–85, acetate was widely used as the main dialysate buffer, but it was virtually abandoned in favor of bicarbonate because acetate-buffered dialysis was associated with more frequent side effects, including hypoxemia and intradialytic hypotension. Nevertheless, concerns remain that even small amounts of acetate generated from acetic acid may exert cardio depressant and unfavorable metabolic effects.

### Citric acid as an alternative acidifying agent

Citric acid was therefore introduced as an alternative acidifying agent. After conversion to citrate, it chelates calcium and magnesium, reduces membrane fouling, and may decrease anticoagulation requirements. The use of Cit-D (2.4 mEq/l) resulted in noninferior dialyzer clearance despite a 20%–33% reduction in heparin dose compared with Acet-D [45]. Switching from Acet-D to Cit-D during online HDF reduced heparin requirements by 17% [46]. The citrate concentrations generated during routine dialysis remain far below those required for systemic anticoagulation, which would require concentrations ∼5-fold higher [47].

### Calcification propensity

Beyond anticoagulation sparing, Cit-D may exert independent anti-inflammatory and metabolic benefits [48–51]. Owing to its calcium-chelating properties, Cit-D may also reduce calcification propensity. This has been evaluated using the T_50_ test, which assesses the transformation time of primary into secondary calciprotein particles (CPP) *in vitro*. Primary CPP are composed of fetuin-A, calcium, and phosphate combined in a colloidal particle. Their conversion into hydroxyapatite-containing secondary CPPs likely contributes to vascular calcification. Thus, a shorter T_50_ reflects higher calcification propensity. Reduced T_50_ values are associated with increased all-cause mortality in patients on HD [52].

Two studies demonstrated an improvement in T_50_, after switching from Acet-D to Cit-D. In a randomized cross-over study evaluating three dialysate prescriptions over 1 week, we observed a significantly longer T_50_ with Cit-D combined with DCa 1.50 (Cit-D–DCa 1.50) compared to Acet-D–DCa 1.50. Predialysis T_50_ increased during treatment with Acet-D–DCa 1.25 and Cit-D–DCa 1.50 but remained stable with Acet-D–DCa 1.50. Lorenz *et al*. [53] switched 77 patients from Acet-D to Cit-D for 3 months, of whom 44 were subsequently switched back to Acet-D. T_50_ increased significantly after conversion to Cit-D and remained elevated after switching back to Acet-D [53].

### Clinical outcomes

Whether these biological effects translate into improved clinical outcomes remains uncertain (Table 2). In a retrospective study, use of Cit-D was associated with slower progression of aortic calcification score over a 1-year period compared with Acet-D [54]. However, the effects of Cit-D on vascular calcifications remain uncertain, as a crossover study unexpectedly reported more rapid progression of breast arterial calcification with Cit-D than with Acet-D despite no meaningful differences in mineral metabolism or dialysis delivery [55]. Initial concerns had been raised regarding a possible increase in mortality with Cit-D [56]. However, three observational trials found no significant differences in mortality between Cit-D and Acet-D [56–59], whereas one reported a lower mortality with Cit-D compared to Acet-D [60].

**Table 2:** Potential clinical implications of acetic acid and citric acid dialysates.

Dialysate acidification	Potential advantages	Potential concerns	Potential clinical indications
**Acetic-acid (Acet-D)**	Large experience	Residual acetate exposure and higher heparin requirements Greater calcium loading and less favorable serum calcification propensity than Cit-D at the same nominal DCa	Present reference solution
**Citric acid (Cit-D)**	Lower anticoagulation requirements Possible reduction in membrane fouling Reduced calcium loading and improved calcification propensity	More negative Ca/Mg balance; Hypotension or muscle cramps in susceptible patients No proven superiority for hard clinical outcomes Uncertainty about clinical effects on vascular calcification	High bleeding risk or a need to reduce heparin exposure High heparin requirements

There has also been concern that the larger dialysate/ substitution volumes used during online HDF could increase citrate exposure and thereby enhance side effects. In a randomized crossover study including predominantly HDF patients, the use of Cit-D with DCa 1.50 was associated with a higher incidence of cramps and fatigue, although no significant differences in PiCa or alkalosis were observed [61].

### Effects on CaMB

Cit-D dialysate also exerts different effects on CaMB as compared to dialysate with Acet-D. This is mainly explained by the ability of citrate to chelate calcium, with subsequent removal of calcium–citrate complexes during dialysis. In our study, Cit-D with DCa of 1.50 resulted in a negative mean CaMB (−80 mg/treatment) comparable to that observes with Acet-D–DCa of 1.25 (−96 mg) whereas Acet-D–DCa 1.50 mmol/l produced an overall positive CaMB (+5.7 mmol) [40]. To achieve an equivalent CaMB, the DCa during Cit-D should be ∼0.15 mmol/l higher than that with Acet-D [62]. The combination of Cit-D with DCa 1.25 is likely to induce a markedly negative CaMB, with effects on calcium homeostasis comparable with Acet-D at a DCa below 1.25 mmol/l. In our opinion, when Cit-D is used, the combination with DCa 1.50 therefore appears to be the most appropriate option for majority of patients.

### Effects on intradialytic blood pressure and QT interval

Potential drawbacks of Cit-D include QT interval prolongation related to reductions in PiCa. In our study, QTc increased significantly during dialysis with both Cit-D–DCa 1.50 and Acet-D–DCa 1.25 but not with Acet-D–DCa 1.50. However, these effects were relatively modest and are likely to be clinically relevant mainly in patients with a pre-existing prolonged QT interval [63]. Possibly due to an effect on PiCa, we also observed a significantly lower nadir systolic blood pressure with Cit-D–DCa 1.50 compared to Acet-D–DCa 1.50 [12].

### Clinical perspective

Overall, although Cit-D may offer potential advantages, including reduced calcification propensity, improved dialysis efficacy, and lower anticoagulation requirements, convincing evidence for long-term clinical benefit is lacking. Large-scale randomized trials are needed to define its role in dialysis therapy.

## MAGNESIUM

### Magnesium as an inhibitor of calcification and modulator of cardiac risk

Magnesium (Mg) is increasingly recognized as a key endogenous inhibitor of vascular calcification, and low plasma Mg levels have been associated with increased cardiovascular mortality [64]. An observational study in 142.555 patients on HD showed a J-shaped relation between plasma Mg and all-cause mortality, with far higher odds ratio at the lower levels. The serum magnesium level associated with the lowest mortality was observed with mild hypomagnesemia (∼2.7 mg/dl = 1.1 mmol/l) [65].

Conventional HD using a standard dialysate magnesium concentration (DMg) of 0.50 mmol/l (DMg 0.50) (=1.0 mEq/l) generally induces a reduction in plasma Mg levels [66]. Therefore, increasing DMg has been proposed as a strategy to improve cardiovascular outcomes, although evidence on hard clinical outcomes remains limited. In an observational trial including 75 patients, cardiovascular mortality was lower in patients with a DMg of 0.75 mmol/l (DMg 0.75) compared with matched controls receiving DMg 0.50 [67]. In addition, a randomized cross-over study showed that increasing DMg improved arterial stiffness and reduced predialysis systolic blood pressure without increasing the risk of intradialytic hypotension [68].

With regard to calcification propensity, Bressendorf *et al*., using an assay other than T_50_ in a post hoc analysis of a randomized clinical trial, demonstrated that total, primary, and secondary CPP levels, as well as the inflammatory markers tumor necrosis factor α (TNF-α) and interleukin 6 (IL-6) decreased 28 days after conversion from DMg 0.50 to 1.0 mmol/l in the intervention group, whereas these parameters remained stable in the control group [69].

Thus, both increasing DMg and replacing Acet-D with Cit-D appear to improve calcification propensity and vascular wall characteristics. However, somewhat conflicting results were reported in the CitMag study, in which the combination of DMg 0.75 and Cit-D over 3 weeks did not improve CPP formation with DMg 0.50 [70].

These discrepancies may partly relate to differences in study design and timing of the measurements. In the CitMag study, T_50_ was measured in the predialysis period, whereas in the study by ter Meulen *et al*. the most pronounced effects of Cit-D were observed immediately postdialysis, which is biologically plausible given the relatively short half-life of citrate. Although predialysis T_50_ levels are probably more relevant for long-term calcification risk, intradialytic changes may also contribute to vascular calcification over time through repeated alterations in calcium kinetics [31, 32]. Importantly, Cit-D also chelates Mg, and the use of Cit-D may therefore promote a negative magnesium balance unless DMg is appropriately adjusted.

### Clinical perspective

Overall, current evidence supports magnesium as an important therapeutic target for reducing calcification propensity and improving cardiovascular health in HD patients. Irrespective of its effects on calcium propensity, a higher DMg may be advisable when using Cit-D. Pending outcome trials, DMg 0.75 may be considered to minimize negative magnesium balance and potentially improve cardiovascular protection (Table 3).

**Table 3:** Potential clinical implications of DMg.

DMg	Potential advantages	Potential concerns	Potential clinical indications
**0.50 mmol/l** *Standard DMg*	Standard concentration in commonly used dialysate	Negative magnesium balance and effects of hypomagnesemia	Most commonly used
**0.75 mmol/l**	Associated with lower cardiovascular mortality? Improved arterial stiffness Reduced predialysis systolic blood pressure	Limited outcome evidence	Patients with hypomagnesemia Potential for routine use, particularly with low or declining plasma magnesium or during Cit-D
**1.0 mmol/l**	Possibly lower calcification propensity Reduced TNF-α and IL-6	Risk for hypermagnesemia leading to arrythmias? Limited evidence	Research or selected clinical use

## CONCLUSION

The effects of DCa, DMg, and the acidifying component on mineral balance, calcification propensity, and cardiovascular pathophysiology are strongly interrelated. Cit-D offers several theoretical advantages over Acet-D, whereas observational studies have reported inconsistent findings regarding its effect on the progression of vascular calcification.

The optimal target for CaMB and its long-term clinical implications remain uncertain. Most of the available data come from observational studies or small interventional studies. Current evidence does not demonstrate a clear outcome difference between DCa 1.25 and 1.50. DCa should, in our opinion, be prescribed in the context of an integrated CKD-MBD management strategy. Considering the potential impact of calcium load on vascular stiffness and calcification, DCa 1.25 appears to be a rational choice when using Acet-D, especially in patients on calcium-containing phosphate binders and those with low PTH levels, whereas the use of DCa 1.50 is rational in those patients at high risk of SCD and in patients with severe hyperparathyroidism or hemodynamic instability. Individualized prescription and improved assessment of intradialytic mineral balance should be priorities for future research and practice.

## Data Availability

No new data were generated or analyzed in support of this research.
